# A connectomic approach to the lateral geniculate nucleus

**DOI:** 10.1017/S0952523817000116

**Published:** 2017-10-23

**Authors:** JOSH L. MORGAN

**Affiliations:** 1 Department of Ophthalmology and Visual Sciences, Department of Neuroscience, Washington University School of Medicine, Saint Louis, Missouri 63110

**Keywords:** LGN, Connectomics, RGC, Electron microscopy, Lateral geniculate nucleus

## Abstract

Although the core functions and structure of the lateral geniculate nucleus (LGN) are well understood, this core is surrounded by questions about the integration of feedforward and feedback connections, interactions between different channels of information, and how activity dependent development restructures synaptic networks. Our understanding of the organization of the mouse LGN is particularly limited given how important it has become as a model system. Advances in circuit scale electron microscopy (cellular connectomics) have made it possible to reconstruct the synaptic connectivity of hundreds of neurons within in a circuit the size of the mouse LGN. These circuit reconstructions can reveal cell type-to-cell type canonical wiring diagrams as well as the higher order wiring motifs that are only visible in reconstructions of intact networks. Connectomic analysis of the LGN therefore not only can answer longstanding questions about the organization of the visual thalamus but also presents unique opportunities for investigating fundamental properties of mammalian circuit formation.

## The challenge of complexity

The structure of the nervous system is complex at a level that both experimental and theoretical neuroscience are struggling to come to grips with. Sensory data are processed by networks of many thousands of synaptically connected neurons, but connections between neurons are usually mapped using one or two cells at a time. The pattern of connections within these networks is shaped by every level of neuronal interaction from synaptic geometry to global network activity, but our models of connection probability generally consider only cell type and proximity. Cellular connectomics is an attempt to deal with the daunting complexity neural tissue, not as noise to be averaged out, but as a critical adaptation that must be understood in order to understand the biological origins of behavior.

One of the current challenges of ‘connectomics’ is that the term is used to refer to several distinct big data approaches to neuroanatomy that differ significantly in their techniques and questions. One connectomic approach is to develop better frameworks for compiling the results from large numbers of functional and anatomical experiments into integrated models of connectivity. Large scale examples of this approach include the Human Connectome Project (Glasser et al., 2016) and the Human Brain Project (Amunts et al., 2016). The second approach, critical to capturing the higher order organization of neural networks, is to develop tools that allow for direct observation of intact cellular networks (Morgan & Lichtman, 2013). Such techniques include scaling up calcium imaging and electron microscopy so that the functional or anatomical connections between thousands of cells can be mapped in the same piece of tissue.

The lateral geniculate nucleus (LGN) of the thalamus is ideally situated both to be understood by new cellular connectomic approaches and to contribute to our understanding of how networks of neurons process information. On the one hand, the LGN is a small, well-characterized piece of tissue with a single-output cell type and a well-defined core function. On the other hand, the LGN is developmentally plastic and its core function, relaying retinal information to the cortex, gains complexity through the interaction of multiple channels of visual information and by a large number of modulatory and feedback connections. Examples of potential LGN targets for connectomic analysis include generating an inventory of cell and synapse types, generating a wiring diagram of how information is shared across visual channels, identifying the level of specificity with which feedback connections are registered to feedforward connections, or mapping how neighboring neurons from the same functional mosaic deploy their synapses relative to one another. Although these issues have been addressed, to some extent, in a rich literature on LGN connectivity (see this issue), the hope is that connectomic techniques can address many of these questions simultaneously using images and models of intact networks.

## More than one wire in the LGN

A piece of tissue that is often referred to as a ‘relay’ might seem like an unlikely piece of the brain in which to study synaptic wiring. There are many examples of thalamocortical cell (TC) recordings, where the response properties of TCs appear unchanged from the response properties of the retinal ganglion cells (RGCs) that drive them (Smith et al., 1990; Sincich et al., 2007). Such responses are consistent with a simple one-to-one (one RGC to one TC) wiring plan in which the role of the LGN is to provide a way for the cortex to disconnect from the retina. However, the LGN is synaptically overpowered for this function. Up to 93 percent (depending on the species) of the synapses in the LGN are not between RGCs and TCs (Van Horn et al., 2000), but instead come from excitatory feedback from the cortex, feedback inhibition from the reticular nucleus, local feedforward inhibitory connections, input from optic tectum, and input from the brainstem (for review of TC synaptology, see Sherman & Guillery, 2004). Rather than being diffuse or random, these nonretinal synapses are often retinotopic (Ichida & Casagrande, 2002; Ichida et al., 2014), channel specific (Briggs & Usrey, 2009; Ichida et al., 2014), and can be targeted down to the level of individual synapses (Famiglietti & Peters, 1972). Instead of being structured as a simple ON/OFF relay, the LGN would be better described as converging arrays of transistors that constitute the front end of a set of thalamocortical loops.

However, this analogy is also incomplete because, even among the synaptic connections between the retina and TCs, only a subset appears consistent with the function of a gated relay, i.e., one-to-one RGC to TC connectivity. Based on physiological data, one-to-one connections appear to be common in the primate, fovea dominated, LGN (Sincich et al., 2007), but the motif is found in only a minority of TCs in the cat (Cleland et al., 1971; Mastronarde, 1992) and is rarely observed in rodents (Hong et al., 2014; Weyand, 2016). Recent results in the mouse, in particular, reveal the convergence of different channels of visual information and the generation of new response properties (Marshel et al., 2012; Piscopo et al., 2013; Rompani et al., 2017).

The LGN, therefore, is a stage of visual processing that shares a basic wiring plan with the previous retinal stages of visual processing, but which also introduces new circuit properties. Like in the retina, in the LGN, there is a clear core of feedforward excitatory transmission of visual information that is spatially and temporally refined by local inhibitory neurons. Like the retina, the feedforward core of the LGN is anatomically organized according to visual space and visual channel and it exhibits channel-specific levels of divergence and convergence. However, unlike in the retina, the feedforward transmission is heavily modulated by other regions of the brain. Also unlike in the retina, most aspects of the feedforward circuitry are heavily dependent on developmental activity patterns. In the retina, disrupting synaptic transmission alters the numbers of synapses formed between different classes of neurons (Kerschensteiner et al., 2009; Morgan et al., 2011), but the basic wiring plan of the retina appears to be defined before the synaptic transmission begins (parallel arrays of bipolar cells and stratified dendritic and axonal mosaics). Altering developmental activity patterns has a much more dramatic effect in the LGN, where RGC axons face the challenge of reconstructing spatial and synaptic relationships after being bundled together in the optic tract. Nearly, every property of LGN circuitry—receptive field size, segregation of channels, and segregation of inputs for different eyes—is significantly disrupted by disruptions of developmental activity patterns (Hahm et al., 1991; Hong & Chen, 2011).

It is the LGN’s balance between structural/functional stereotypy and developmental plasticity that makes it a particularly appealing target tissue for connectomic analysis. When neurons change their connectivity in response to the firing patterns of their synaptic neighbors, higher order connectivity patterns can emerge that could not be predicted simply from knowing the type and position of two neurons. In the simplest Hebbian form, whether or not an axon will remain connected to a given target cell will depend on whether the other inputs to the target neuron cause it to fire with the first axon. In the LGN, this sort of Hebbian plasticity has been strongly implicated in driving the segregation of RGC inputs coming from different eyes (Torborg et al., 2005; Butts et al., 2007). At the synaptic level, these sorts of processes should generate identifiable wiring motifs. For instance, the observation that two given axons formed stable connections on one target cell would predict that those same axons should be unusually likely to form stable connections on other target cells. More generally, in networks shaped by activity, the probability of cell A and B being connected can be dependent on whether cell C and B are connected. In order to understand how network activity becomes network structure (developmental and adult learning), we need plastic, but decipherable, neural networks like the LGN to be mapped using techniques that can reveal higher order wiring patterns.

## Mapping LGN connectivity with electron microscopy

Electron microscopy (EM) has been used extensively to study the synaptic organization of the LGN. Studies that have combined EM with degeneration tracing, immunostaining, Golgi staining, and biocytin labeling have characterized ultrastructural identifiers for most of the cell types present in the LGN. For example, RGC inputs can be identified by their light mitochondria, round large and pale synaptic vesicles, and by their targeting of proximal TC dendrites (Szentagothai et al., 1966; Famiglietti & Peters, 1972). Tectal inputs resemble RGC inputs, but can be distinguished by their dark mitochondria (Bickford et al., 2015). Inhibitory reticular nucleus axons target the spiny distal dendrites of TCs with boutons that have small vesicle and dark mitochondria (Wang et al., 2001). Despite wide variation in the gross organization of the LGN across mammalian species, the same basic cell types and synaptic motifs are easily recognized in electron micrographs from rodents, cats, and primates (Colonnier & Guillery, 1964; Guillery, 1969; Guillery & Colonnier, 1970; Rafols & Valverde, 1973; Wilson, 1989). One easily identifiable higher order motif is the synaptic triad that can be found at most of the synapses between RGCs and TCs. The triad is formed by a local inhibitory neuron process that both receives input from the presynaptic RGC and goes on to innervate the postsynaptic TC (Famiglietti & Peters, 1972; Ohara et al., 1983; Hamori et al., 1991). Because these synapses often occur within a few micrometers of each other, this motif can be identified in a 2D electron micrograph. The synapse level feedforward inhibition provided by these triad synapses is thought to act as temporal sharpening filter (Blitz & Regehr, 2005). However, these triad synapses are also part of a larger glial encapsulated synaptic structure termed as glomerulus (Szentagothai, 1963) in which many RGC and local inhibitory neuron boutons cluster around the proximal dendrites of TCs. The fact that multiple RGCs converge within these glomerular clusters (Hamos et al., 1987; Hammer et al., 2015; Morgan et al., 2016) suggests that the local feedforward inhibition might be part of more a complex dendritic computation such as coincidence detection. Making sense of these sorts of complex synaptic structures requires being able to identify the cohorts of neurons that participate in them.

The biggest limit to reconstructing circuits with electron microscopy is that ultrathin sectioning and nanometer resolution imaging historically meant that only small volumes could be reconstructed. One approach to overcoming this limitation has been to target a neuron using a label that is visible both optically and in an electron micrograph. Using this method, Hamos and Sherman (Hamos et al., 1987) were able to identify and reconstruct the target TCs innervated by an horseradish peroxidase labeled type X RGC axonal branch. They found that the axon passed by a large number of potential TC partners to selectively form synapses on four target cells. The connectivity between the labeled axon and the four identified target cells was remarkable in its diversity. The X axon synapsed with three morphologically distinct X TCs and one Y TC. Among these X TCs, the labeled axon accounted for 100% of the inputs onto one cell, 91% on another, and only 33% on the third. In their 1987 article, Hamos and Sherman state that the major limitation of their study was that it was labor intensive and time consuming. The effort required to reconstruct a single axonal arbor meant that the diversity in connections that they observed would be difficult to confirm: “it will never be practical to obtain such analyses for large populations of afferent axons”.

Although it still may not be feasible to acquire hundreds of circuit scale EM datasets in which a single-labeled RGC axon is traced in each, it is now possible to acquire a single EM dataset in which hundreds of unlabeled RGC axons can be traced. Tracing large numbers of unlabeled neurons requires that large regions of neuropil are digitized at sufficient resolution that nonspecific membrane stains (such as osmium, lead, and uranium) are sufficient for following thin processes over long distances. Modern data storage and image processing capabilities are critical to this endeavor because acquiring high-resolution three-dimensional volumes on the hundred micrometer scale requires terabytes of data storage; the millimeter scale requires petabytes. Advances in data handling capabilities, electron microscopes, and sectioning techniques have allowed for a proliferation of circuit scale 3D EM techniques including block face imaging (Leighton, 1981; Helmstaedter et al., 2013; Hayworth et al., 2015), high-throughput transmission EM (Bock et al., 2011), and scanning EM-based tape collection ultramicrotomy (Schalek et al., 2011). Most of these techniques involve a significant amount of automation in the acquisition and processing of EM images. Among the largest of EM datasets that have been produced in recent years, the mammalian visual system is well represented. Large scale EM reconstructions have been performed on the rabbit (Anderson et al., 2011) and mouse retina (Briggman et al., 2011; Helmstaedter et al., 2013), mouse LGN (Morgan et al., 2016), and mouse visual cortex (Bock et al., 2011; Lee et al., 2016). Each of these studies has significant limitations due to tissue staining, traceability, and/or reconstruction size. However, in each case, important biological observations were obtained and there is a clear path forward for improving techniques. In particular, the ability to fully utilize these datasets depends on improvements in applying machine vision to EM data, an area in which rapid progress is being made (Arganda-Carreras et al., 2015; Kaynig et al., 2015; Januszewski et al., 2016; Zeng et al., 2017).

Critically, the quantity of data that can now be collected in a single piece of tissue has led to qualitative changes in the kinds of questions that can be asked. Because every cell and organelle is labeled and imaged in a 3D EM volume, the resulting dataset is essentially a digitization of a piece of fixed tissue. Within such a dataset, it is possible to map intact networks of hundreds of synaptically connected neurons. These networks contain large amounts of traditional connectivity data; i.e., which cell types connect to which cell types and how many inputs and outputs each neuron has. These datasets also make it possible to trace networks of hundreds of synaptically connected neurons and search for higher order connectivity motifs. For instance, within a population of neurons of the same cell type it is possible to identify cohorts of axons that preferentially target the same postsynaptic neurons. Finally, electron micrographic connectivity maps have the advantage that each node in the acquired network represents a rich structural dataset that includes cell morphology, the location of synapses, and intracellular ultrastructure. This rich structural data mean that each fully reconstructed cell in a network can be reliably classified as to cell type and that ultrastructural details about individual synapses can be related to their role in the larger network.

**Fig. 1.**
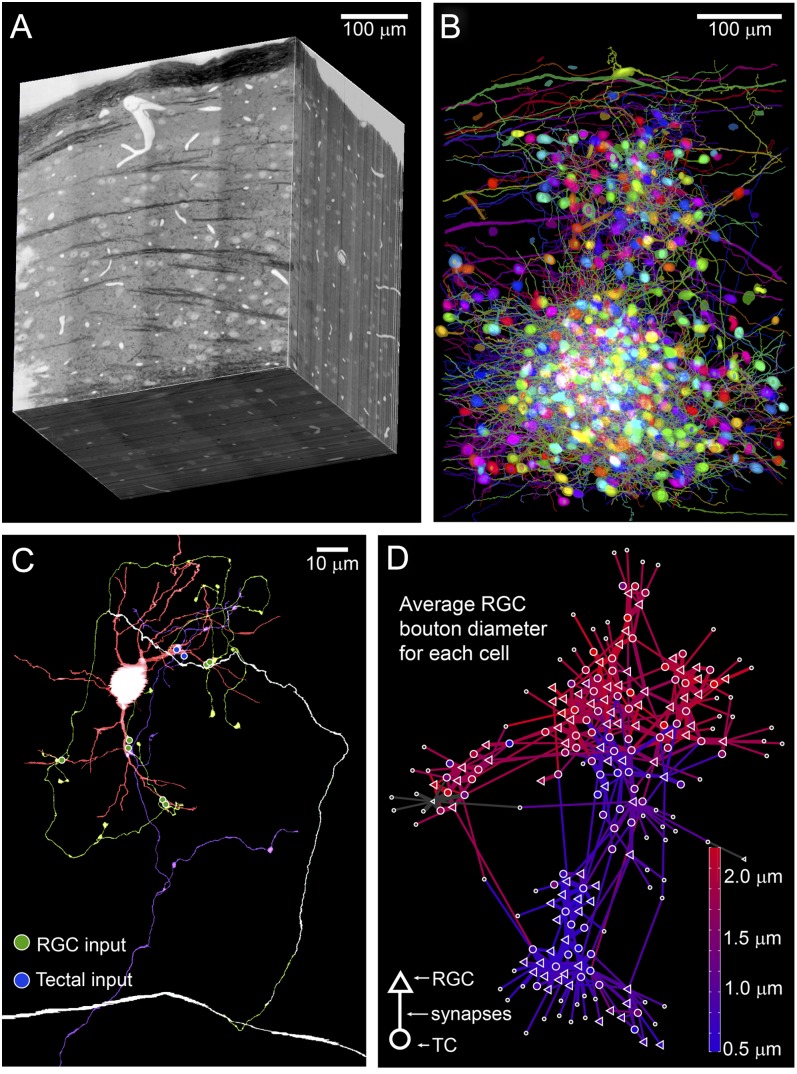
Reconstructing connections within an electron microscopy image volume of a P32 mouse LGN. (**A**) Aligned EM volume of mouse LGN. Optic tract is visible as a dark band at the surface. (**B**) Targeted manual segmentations of about 1% of imaged voxels from panel A. (**C**) Example tracing from EM volume in panel A in which an RGC axon (green) and a tectal axon (blue) innervate the proximal dendrites (red) of a TC. The myelin around the RGC axon is labeled white. (**D**) A force directed model of a subset of the traced neurons from panel B demonstrates that large bouton forming and small bouton forming RGCs sometimes segregate and sometimes mix together in their innervation of TCs. Synaptic connections (lines) are used to pull RGCs (trangles) and TCs (circles) into clusters of highly interconnected neurons. Neurons are color coded according to the average diameter of RGC boutons associated with the TC or RGC.

One of the largest publicly available circuit scale EM volumes is an image volume of the mouse dorsal LGN (Morgan et al., 2016) (Fig. 1A). This dataset encompasses the full depth of a P32 mouse LGN (500 *µ*m), a little less than half the width of the LGN (400 *µ*m) and about a third of the rostro-caudal extent of the LGN (300 *µ*m) (raw voxel size = 4 × 4 × 30 nm). Contained within this volume are thousands of TC cell bodies and hundreds of thousands of synapses. The initial publication of this dataset included a manual segmentation of ∼1% of the 100 trillion imaged voxels (Fig. 1B and 1C). These tracings consist primarily of a network of synaptically interconnected RGCs and TCs. The majority of these tracings were performed manually using VAST (by Daniel Berger) and an aligned image volume that was down sampled to 16 × 16 × 30 nm voxel size. This down-sampled image volume, the VAST tracing software, and the published segmentations are available at https://software.rc.fas.harvard.edu/lichtman/LGN/. In addition, the collection of Matlab code (NautilusAnalysis) that was used to perform most of the rendering and analysis of the LGN data is available on this site. Any researcher needing ultrastructural, morphological or connectivity data for the mouse LGN is free to download and use the data. Work on improving this dataset is ongoing and it is hoped that a full resolution aligned dataset and annotation database will be available in the future.

The results of our initial tracings of this LGN EM volume were similar in many ways to the results observed by Hamos et al. (1987). We found that a single RGC could innervate a diverse population of TCs; TCs with different dendritic and synaptic structures. We were also able to determine that while some TCs were dominated by a single morphological type of RGC, other TCs were innervated by multiple types of RGCs. Although visual channels mixed and split in the mouse LGN, connectivity was not random. We observed specificity in RGC wiring both at the network level (preferred TC target populations) and at the microcircuit level (cohorts of RGCs targeting particular TC dendrites). We referred to this circuit organization as fuzzy, because although we could define clusters of cells by connectivity and ultrastructure, the boundaries between these groups were indistinct and individual neurons could be said to be members of multiple groups (Fig. 1D).

We were particularly surprised at the wide range we observed in the number of RGC axons that innervated TCs. Based on both anatomical and electrophysiological estimates of RGC convergence onto TCs (Cleland et al., 1971; Hamos et al., 1987; Mastronarde et al., 1991; Chen & Regehr, 2000; Hong & Chen, 2011), we expected TCs to be innervated by 1–4 strong inputs and a few weaker inputs. We did observe some TCs in the mouse that were innervated by only a few very strong RGC inputs (each axon forming many large multisynaptic boutons) and several weak inputs. However, many other TCs appeared to be innervated by dozens of RGC axons. To some extent, this high convergence might be attributed to the young age (P32) of the reconstructed mouse or to the incomplete tracing of RGC axons that leave the imaged volume (Chen et al., 2016). However, similarly high convergence of mouse RGCs onto TCs have also been observed in using brainbow identification of RGC inputs (Hammer et al., 2015) and using trans synaptic tracing to the retina (Rompani et al., 2017). Part of the explanation for the high convergence in mouse relative to the rest of the mammalian LGN literature is likely to be simply species specific difference in visual processing (Chen et al., 2016). Until techniques that reveal the complete complement of synaptic partners are applied to a wide range of species and cell types, it is unclear how much these apparent differences in connectivity reflect differences in species *vs.* experimental scale, targets, or techniques. As the speed at which circuit scale EM volumes can be acquired and analyzed increases, it is hoped that such connectivity maps will become a standard resource for most model neural circuits.

## An LGN connectome

What would constitute a completed LGN connectome? A complete LGN connectome should include an inventory of all of the types and variations of LGN TCs and inhibitory cells as wells as all of the subtypes of axons converging on the LGN from the retina, cortex, reticular nucleus, optic tectum, and brainstem. We would know how each type deploys its synapses relative to other types of neurons and how each neuron deploys its synapses relative to other neurons of the same type (convergence, divergence, spatial mapping, competition, and coordination). We would have sufficient data on the size, topology, and cell type-specific physiology of all of these synapses such that reasonable estimates local synaptic interactions and cell wide integration can be made. All of this data would be integrated into a single model of the LGN which can track the flow of information at every level from subcellular microcircuits to global network activity. Ideally, we would have such a model for multiple species, ages, and disease states. The success of such a model would be in large part measured in whether it can make physiologically and behaviorally meaningful predictions.

To the extent that such a model can be produced, it will depend on combining both kinds of connectomic approaches: directly acquiring dense cellular network data and combining different datatypes into a single integrated model. Integrating different datatypes is easiest, of course, when different types of data can be acquired in the same piece of tissue. In the case of circuit scale electron microscopy, there are a number of examples of combining calcium imaging (Lee et al., 2016) or molecular characterization (Anderson et al., 2011) of tissue with large scale EM. However, one of the benefits of generating such an integrated model in the LGN is that there is sufficient stereotypy in the relationship between structure and function that morphologically defined subtypes and retinotopic mapping can be used to anchor many different kinds of datasets together.

There are many important questions that such a model might be able to answer, but one of the most fundamental is, “can we build a functional neural circuit from the ground up and, if not, why not?” What are the critical developmental or physiological rules that would be required to animate such a structural model? At the moment, there is a gulf of circuit complexity between our understanding of how cells process information and our understanding of how brains produce behavior. Big data connectomics approaches are an attempt to bridge that gap.

## References

[ref1] Amunts K., Ebell C., Muller J., Telefont M., Knoll A. & Lippert T. (2016). The human brain project: Creating a European research infrastructure to decode the human brain. Neuron 92, 574–581. 27809997 10.1016/j.neuron.2016.10.046

[ref2] Anderson J.R., Jones B.W., Watt C.B., Shaw M.V., Yang J.H., Demill D., Lauritzen J.S., Lin Y., Rapp K.D., Mastronarde D., Koshevoy P., Grimm B., Tasdizen T., Whitaker R. & Marc R.E. (2011). Exploring the retinal connectome. Molecular Vision 17, 355–379. 21311605 PMC3036568

[ref3] Arganda-Carreras I., Turaga S.C., Berger D.R., Ciresan D., Giusti A., Gambardella L.M., Schmidhuber J., Laptev D., Dwivedi S., Buhmann J.M., Liu T., Seyedhosseini M., Tasdizen T., Kamentsky L., Burget R., Uher V., Tan X., Sun C., Pham T.D., Bas E., Uzunbas M.G., Cardona A., Schindelin J. & Seung H.S. (2015). Crowdsourcing the creation of image segmentation algorithms for connectomics. Frontiers in Neuroanatomy 9, 142. 26594156 10.3389/fnana.2015.00142 PMC4633678

[ref4] Bickford M.E., Zhou N., Krahe T.E., Govindaiah G. & Guido W. (2015). Retinal and tectal “driver-like” inputs converge in the shell of the mouse dorsal lateral geniculate nucleus. Journal of Neuroscience 35, 10523–10534. 26203147 10.1523/JNEUROSCI.3375-14.2015 PMC4510292

[ref5] Blitz D.M. & Regehr W.G. (2005). Timing and specificity of feed-forward inhibition within the LGN. Neuron 45, 917–928. 15797552 10.1016/j.neuron.2005.01.033

[ref6] Bock D.D., Lee W.C., Kerlin A.M., Andermann M.L., Hood G., Wetzel A.W., Yurgenson S., Soucy E.R., Kim H.S. & Reid R.C. (2011). Network anatomy and *in vivo* physiology of visual cortical neurons. Nature 471, 177–182. 21390124 10.1038/nature09802 PMC3095821

[ref7] Briggman K.L., Helmstaedter M. & Denk W. (2011). Wiring specificity in the direction-selectivity circuit of the retina. Nature 471, 183–188. 21390125 10.1038/nature09818

[ref8] Briggs F. & Usrey W.M. (2009). Parallel processing in the corticogeniculate pathway of the macaque monkey. Neuron 62, 135–146. 19376073 10.1016/j.neuron.2009.02.024 PMC2789995

[ref9] Butts D.A., Kanold P.O. & Shatz C.J. (2007). A burst-based “Hebbian” learning rule at retinogeniculate synapses links retinal waves to activity-dependent refinement. PLoS Biology 5, e61. 17341130 10.1371/journal.pbio.0050061 PMC1808114

[ref10] Chen C., Bickford M.E. & Hirsch J.A. (2016). Untangling the web between eye and brain. Cell 165, 20–21. 27015304 10.1016/j.cell.2016.03.010 PMC6193467

[ref11] Chen C. & Regehr W.G. (2000). Developmental remodeling of the retinogeniculate synapse. Neuron 28, 955–966. 11163279 10.1016/s0896-6273(00)00166-5

[ref12] Cleland B.G., Dubin M.W. & Levick W.R. (1971). Sustained and transient neurones in the cat’s retina and lateral geniculate nucleus. Journal of Physiology 217, 473–496. 5097609 10.1113/jphysiol.1971.sp009581 PMC1331787

[ref13] Colonnier M. & Guillery R.W. (1964). Synaptic organization in the lateral geniculate nucleus of the monkey. Zeitschrift fuer Zellforschung und Mikroskopische Anatomie 62, 333–355. 10.1007/BF00339284 14218147

[ref14] Famiglietti E.V. Jr. & Peters A. (1972). The synaptic glomerulus and the intrinsic neuron in the dorsal lateral geniculate nucleus of the cat. Journal of Comparative Neurology 144, 285–334. 4112778 10.1002/cne.901440304

[ref15] Glasser M.F., Smith S.M., Marcus D.S., Andersson J.L., Auerbach E.J., Behrens T.E., Coalson T.S., Harms M.P., Jenkinson M., Moeller S., Robinson E.C., Sotiropoulos S.N., Xu J., Yacoub E., Ugurbil K. & Van Essen D.C. (2016). The human connectome project’s neuroimaging approach. Nature Neuroscience 19, 1175–1187. 27571196 10.1038/nn.4361 PMC6172654

[ref16] Guillery R.W. (1969). The organization of synaptic interconnections in the laminae of the dorsal lateral geniculate nucleus of the cat. Zeitschrift fuer Zellforschung und Mikroskopische Anatomie 96, 1–38. 10.1007/BF00321474 5772028

[ref17] Guillery R.W. & Colonnier M. (1970). Synaptic patterns in the dorsal lateral geniculate nucleus of the monkey. Zeitschrift fuer Zellforschung und Mikroskopische Anatomie 103, 90–108. 10.1007/BF00335403 4983695

[ref18] Hahm J.O., Langdon R.B. & Sur M. (1991). Disruption of retinogeniculate afferent segregation by antagonists to NMDA receptors. Nature 351, 568–570. 1675433 10.1038/351568a0

[ref19] Hammer S., Monavarfeshani A., Lemon T., Su J. & Fox M.A. (2015). Multiple retinal axons converge onto relay cells in the adult mouse thalamus. Cell Reports 12, 1575–1583. 26321636 10.1016/j.celrep.2015.08.003 PMC5757867

[ref20] Hamori J., Pasik P. & Pasik T. (1991). Different types of synaptic triads in the monkey dorsal lateral geniculate nucleus. Journal für Hirnforschung 32, 369–379. 1779135

[ref21] Hamos J.E., Van Horn S.C., Raczkowski D. & Sherman S.M. (1987). Synaptic circuits involving an individual retinogeniculate axon in the cat. Journal of Comparative Neurology 259, 165–192. 3584556 10.1002/cne.902590202

[ref22] Hayworth K.J., Xu C.S., Lu Z., Knott G.W., Fetter R.D., Tapia J.C., Lichtman J.W. & Hess H.F. (2015). Ultrastructurally smooth thick partitioning and volume stitching for large-scale connectomics. Nature Methods 12, 319–322. 25686390 10.1038/nmeth.3292 PMC4382383

[ref23] Helmstaedter M., Briggman K.L., Turaga S.C., Jain V., Seung H.S. & Denk W. (2013). Connectomic reconstruction of the inner plexiform layer in the mouse retina. Nature 500, 168–174. 23925239 10.1038/nature12346

[ref24] Hong Y.K. & Chen C. (2011). Wiring and rewiring of the retinogeniculate synapse. Current Opinion in Neurobiology 21, 228–237. 21558027 10.1016/j.conb.2011.02.007 PMC3099477

[ref25] Hong Y.K., Park S., Litvina E.Y., Morales J., Sanes J.R. & Chen C. (2014). Refinement of the retinogeniculate synapse by bouton clustering. Neuron 84, 332–339. 25284005 10.1016/j.neuron.2014.08.059 PMC4322918

[ref26] Ichida J.M. & Casagrande V.A. (2002). Organization of the feedback pathway from striate cortex (V1) to the lateral geniculate nucleus (LGN) in the owl monkey (*Aotus trivirgatus*). Journal of Comparative Neurology 454, 272–283. 12442318 10.1002/cne.10441

[ref27] Ichida J.M., Mavity-Hudson J.A. & Casagrande V.A. (2014). Distinct patterns of corticogeniculate feedback to different layers of the lateral geniculate nucleus. Eye and Brain 2014, 57–73. 25892906 10.2147/EB.S64281 PMC4399558

[ref28] Januszewski M.M-S.J., Li P., Korfeld J., Denk W. & Jain V. (2016). arXiv:1611.00421. arXiv.

[ref29] Kaynig V., Vazquez-Reina A., Knowles-Barley S., Roberts M., Jones T.R., Kasthuri N., Miller E., Lichtman J. & Pfister H. (2015). Large-scale automatic reconstruction of neuronal processes from electron microscopy images. Medical Image Analysis 22, 77–88. 25791436 10.1016/j.media.2015.02.001 PMC4406409

[ref30] Kerschensteiner D., Morgan J.L., Parker E.D., Lewis R.M. & Wong R.O. (2009). Neurotransmission selectively regulates synapse formation in parallel circuits *in vivo*. Nature 460, 1016–1020. 19693082 10.1038/nature08236 PMC2746695

[ref31] Lee W.C., Bonin V., Reed M., Graham B.J., Hood G., Glattfelder K. & Reid R.C. (2016). Anatomy and function of an excitatory network in the visual cortex. Nature 532, 370–374. 27018655 10.1038/nature17192 PMC4844839

[ref32] Leighton S.B. (1981). SEM images of block faces, cut by a miniature microtome within the SEM—A technical note. Scanning Electron Microscopy 2, 73–76. 7323733

[ref33] Marshel J.H., Kaye A.P., Nauhaus I. & Callaway E.M. (2012). Anterior–posterior direction opponency in the superficial mouse lateral geniculate nucleus. Neuron 76, 713–720. 23177957 10.1016/j.neuron.2012.09.021 PMC3517882

[ref34] Mastronarde D.N., Humphrey A.L. & Saul A.B. (1991). Lagged Y cells in the cat lateral geniculate nucleus. Visual Neuroscience 7, 191–200. 1751414 10.1017/s0952523800004028

[ref35] Mastronarde D.N. (1992). Nonlagged relay cells and interneurons in the cat lateral geniculate nucleus: Receptive-field properties and retinal inputs. Visual Neuroscience 8, 407–441. 1586644 10.1017/s0952523800004934

[ref36] Morgan J.L., Berger D.R., Wetzel A.W. & Lichtman J.W. (2016). The fuzzy logic of network connectivity in mouse visual thalamus. Cell 165, 192–206. 27015312 10.1016/j.cell.2016.02.033 PMC4808248

[ref37] Morgan J.L. & Lichtman J.W. (2013). Why not connectomics? Nature Methods 10, 494–500. 23722208 10.1038/nmeth.2480 PMC4184185

[ref38] Morgan J.L., Soto F., Wong R.O. & Kerschensteiner D. (2011). Development of cell type-specific connectivity patterns of converging excitatory axons in the retina. Neuron 71, 1014–1021. 21943599 10.1016/j.neuron.2011.08.025 PMC3184549

[ref39] Ohara P.T., Lieberman A.R., Hunt S.P. & Wu J.Y. (1983). Neural elements containing glutamic acid decarboxylase (GAD) in the dorsal lateral geniculate nucleus of the rat; immunohistochemical studies by light and electron microscopy. Neuroscience 8, 189–211. 6341876 10.1016/0306-4522(83)90060-x

[ref40] Piscopo D.M., El-Danaf R.N., Huberman A.D. & Niell C.M. (2013). Diverse visual features encoded in mouse lateral geniculate nucleus. Journal of Neuroscience 33, 4642–4656. 23486939 10.1523/JNEUROSCI.5187-12.2013 PMC3665609

[ref41] Rafols J.A. & Valverde F. (1973). The structure of the dorsal lateral geniculate nucleus in the mouse. A Golgi and electron microscopic study. Journal of Comparative Neurology 150, 303–332. 4124620 10.1002/cne.901500305

[ref42] Rompani S.B., Mullner F.E., Wanner A., Zhang C., Roth C.N., Yonehara K. & Roska B. (2017). Different modes of visual integration in the lateral geniculate nucleus revealed by single-cell-initiated transsynaptic tracing. Neuron 93, 1519. 28334614 10.1016/j.neuron.2017.03.009 PMC6381445

[ref43] Schalek R., Kasthuri N. & Hayworth K.J. (2011). Development of high-throughput, high-resolution 3D reconstruction of large-volume biological tissue using automated tape collection ultramicrotomy and scanning electron microscopy. Microscopy and Microanalysis 17, 966–967.

[ref44] Sherman S.M. & Guillery R.W. (2004). Thalamus In Synaptic Organization of the Brain (5th ed.), ed. Shepherd G.M., pp. 311–359. Oxford and New York: Oxford University Press.

[ref45] Sincich L.C., Adams D.L., Economides J.R. & Horton J.C. (2007). Transmission of spike trains at the retinogeniculate synapse. Journal of Neuroscience 27, 2683–2692. 17344406 10.1523/JNEUROSCI.5077-06.2007 PMC6672514

[ref46] Smith E.L. 3rd, Chino Y.M., Ridderh W.H. 3rd, Kitagawa K. & Langston A. (1990). Orientation bias of neurons in the lateral geniculate nucleus of macaque monkeys. Visual Neuroscience 5, 525–545. 2085469 10.1017/s0952523800000699

[ref47] Szentagothai J. (1963). The structure of the synapse in the lateral geniculate body. Acta Anatomica 55, 166–185. 14101379

[ref48] Szentagothai J., Hamori J. & Tombol T. (1966). Degeneration and electron microscope analysis of the synaptic glomeruli in the lateral geniculate body. Experimental Brain Research 2, 283–301. 5957903 10.1007/BF00234775

[ref49] Torborg C.L., Hansen K.A. & Feller M.B. (2005). High frequency, synchronized bursting drives eye-specific segregation of retinogeniculate projections. Nature Neuroscience 8, 72–78. 15608630 10.1038/nn1376 PMC1463890

[ref50] Van Horn S.C., Erisir A. & Sherman S.M. (2000). Relative distribution of synapses in the A-laminae of the lateral geniculate nucleus of the cat. Journal of Comparative Neurology 416, 509–520. 10660881

[ref51] Wang S., Bickford M.E., Van Horn S.C., Erisir A., Godwin D.W. & Sherman S.M. (2001). Synaptic targets of thalamic reticular nucleus terminals in the visual thalamus of the cat. Journal of Comparative Neurology 440, 321–341. 11745627 10.1002/cne.1389

[ref52] Weyand T.G. (2016). The multifunctional lateral geniculate nucleus. Reviews of Neuroscience 27, 135–157. 10.1515/revneuro-2015-0018 26479339

[ref53] Wilson J.R. (1989). Synaptic organization of individual neurons in the macaque lateral geniculate nucleus. Journal of Neuroscience 9, 2931–2953. 2769372 10.1523/JNEUROSCI.09-08-02931.1989 PMC6569706

[ref54] Zeng T., Wu B. & Ji S. (2017). DeepEM3D: Approaching human-level performance on 3D anisotropic EM image segmentation. Bioinformatics 33, 2555–2562. 28379412 10.1093/bioinformatics/btx188 PMC6248556

